# Editorial: Computational methods for pain pharmacology

**DOI:** 10.3389/fpain.2023.1298882

**Published:** 2023-10-19

**Authors:** Jakub Mlost, Mateusz Kucharczyk

**Affiliations:** ^1^ Science for Life Laboratory, Department of Biochemistry and Biophysics, Stockholm University, Stockholm, Sweden; ^2^ Laboratory of Neurophysiology, Department of Biochemical Toxicology, Faculty of Pharmacy, Jagiellonian University Medical College, Krakow, Poland; ^3^ The Wolfson Centre, King’s College London, London, United Kingdom

**Keywords:** computational methods, pain, machine learning, artificial intelligence, big data, data mining

**Editorial on the Research Topic** Computational methods for pain pharmacology

As we venture into the year 2024, it becomes clear that we stand in front of a profound technological revolution. Recent developments in the field of data science and artificial intelligence have now made them apparent parts of our daily life. Advanced technologies are not only reshaping our daily experiences but also revolutionizing how we approach intricate challenges like pain management. The complexity of pain perception, the diversity of underlying mechanisms, and the necessity for tailored pharmacological interventions have fueled a growing interest in employing computational methods to enhance our understanding and treatment of pain.

Two articles in this Research Topic leveraged so-called “big data” to tackle clinically relevant problems. Holmes et al., have used different machine learning (ML) classifiers to differentiate pediatric and young adult subjects with post-traumatic headache (PTH) from healthy controls. Authors used data from different self- or caregiver-report questionnaires that reflect concussion symptoms, mental health, pain experience of the participants, as well as brain imaging data including volume measures of cortical and sub-cortical structures. Abovementioned data allowed researchers to predict PTH diagnosis with 85% accuracy, but more interestingly, they were able to establish the score in Child and Parent Fear of Pain Questionnaire to be the most important predictor of PTH, followed by changes in volumes of several brain areas, implicating their role in the neurobiological mechanism involved in the PTH development. These results highlight how much insight, one can get from the data by using computational tools, such as feature selection algorithms. Similarly, Lai et al., used an *a priori* algorithm to reveal the use patterns of Traditional Chinese Medicine (TCMs) as analgesic drugs in rheumatoid arthritis patients. This study had screened out the potential compatibility combination patterns of drugs for the treatment of rheumatoid arthritis and also set a perfect example of how the use of computational tools can bring better understanding of complex and vague phenomena such as the pharmacology of TCMs and the synergistic effects between various herbs.

This Research Topic also demonstrates how computational approaches are driving the development of personalized pain management strategies. By integrating patient-specific data, such as genetic profiles, physiological parameters, and pain phenotypes, computational models can assist clinicians in selecting the most suitable medications and dosages, optimizing outcomes, and minimizing adverse effects. Van der Wal et al., have employed nociception level monitor (NOL) during operation to dose the analgesics. NOL, based on random forest algorithm, constructed pain score from the following parameters: heart rate, heart rate variability amplitude of the finger photoplethysmogram, skin conductance level and their time derivatives. Whenever the patient reached a threshold pain score, they were given an analgesic. This method was compared with standard procedure, where analgesics are dosed based on hemodynamic response. Authors observed that in the NOL-guided group, fewer patients experienced severe pain after operation and less patients required post-operative pain treatment. Thus, application of NOL or other ML based algorithms for dose optimization may significantly reduce risk of opioid overuse.

Finally, a great proportion of computational work focuses on creating functional models of biological phenomena. Neilan et al., have developed a 3-D computational model of neurons in the central amygdala (CeA) based on the laboratory data. CeA is a heterogenous nucleus including dozens of unique cell types with different functions based on intra-CeA connectivity and localization along the anterior-posterior axis. Generally, neurons expressing protein kinase C-delta (PKCδ) have a pro-nociceptive role in CeA “pain output”, while somatostatin (SST) positive CeA neurons have an anti-nociceptive role. However, there is considerable evidence for expression differences in PKC*δ* and SST as well as other CeA cell-types based on their location within the nucleus. Nociceptive inputs to these cells differ as well. Authors developed the first computational model of PKCδ and SST neurons in the amygdala, based on cell-type physiology data. Their model simulates the behaviors and interactions of individual PKCδ and SST neurons and is capable of capturing morphological features as well as spatial heterogeneity in the location and connectivity of PKCδ and SST neurons throughout the CeA. One of the advantages of the 3-D model is its ability to target cells in different locations within the CeA to investigate the potential for spatially separate populations to differently impact nociception. The value of computational modeling is its ability to address these questions quickly prior to investment in resource-heavy wet lab experiments. Based on the simulations, authors suggest that manipulation of the anterior vs. posterior CeA causes a disproportional impact on nociceptive output from two populations of CeA neurons, a phenomenon recently demonstrated in mice (1).

In conclusion, the research topic “Computational Methods for Pain Pharmacology” underscores the transformative role of computational methods in revolutionizing pain management and research. From unraveling the intricacies of pain neurobiology to accelerating drug discovery and enabling personalized treatments, computational approaches are pushing the boundaries of our understanding and capabilities. As technology continues to evolve and interdisciplinary collaborations flourish, the future holds the promise of more effective, safer, and patient-centric pain relief strategies.
